# Web databases of feather photographs are useful tools for avian morphometry studies

**DOI:** 10.1002/ece3.7600

**Published:** 2021-06-02

**Authors:** Juan E. Malo, Cristina Mata

**Affiliations:** ^1^ Terrestrial Ecology Group (TEG‐UAM) Departamento de Ecología Facultad de Ciencias Universidad Autónoma de Madrid Madrid Spain; ^2^ Centro de Investigación en Biodiversidad y Cambio Global (CIBC‐UAM) Universidad Autónoma de Madrid Madrid Spain

**Keywords:** aspect ratio, bird, feather, measurement error, photogrammetry, size, web database, wing area

## Abstract

1. Wing area, wing loading, and aspect ratio are key variables for studies of avian comparative ecology, despite the complexity of measuring wing characteristics in living and museum specimens. The systematic databases of feather photographs available on the Internet may offer an alternative way of obtaining such morphometric data. Here, we evaluate whether measurements of scanned feathers from web photograph databases may offer reliable estimates of avian morphometry.
2. Published data on wing area were obtained for 317 bird species and feather measurements from web photograph databases for 225 of them. A variable termed “lift generation area,” a proxy for wing area, was calculated for each species on the basis of the mean length of the five distal secondary feathers and wingspan data from literature. The fit between this proposed variable and data extracted from the literature was examined by correlation, employing linear regression to explore the lack of fit among species.
3. “Lift generation area” proved to be highly informative as a proxy for wing area for the study species as a whole (*R* ^2^ > .98). Discrepancies observed between species were strongly negatively associated with the size of the original sample used to calculate wing area (*p* = .001) and, to a lesser extent, with bird size (*p* = .023), but not with aspect ratio. It was also found that the mean value of the mismatch between “lift generation area” and wing area (13.1%) among the study species as a whole was of similar magnitude to that found between sources of bibliographic wing area data for the 64 species for which two published estimates of this variable were available (15.3%).
4. We conclude that measurements made from feather photograph databases are reliable for use in studies of avian comparative ecology, enabling the inclusion of biomechanical parameters of many more species than featured at present.

## Graphical abstract

Wing area and aspect ratio are key variables for studies of avian comparative ecology, despite the complexity of measuring them both in living and in museum specimens. The systematic databases of feather photographs available on the Internet may offer an alternative way of obtaining such morphometric data. Published data on wing area were obtained for 317 bird species, and feather measurements from web photograph databases were found for 225 of them. A variable termed “lift generation area,” a proxy for wing area, was calculated for each species. “Lift generation area” proved to be highly informative as a proxy for wing area for the study species as a whole (*R* ^2^ > .98). We conclude that measurements made from feather photograph databases are reliable for use in studies of avian comparative ecology.

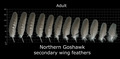

## Resumen

1. Las variables “*wing area,*” *“wing loading”* y “*aspect ratio*” son de gran interés en estudios de ecología comparada de aves, si bien su medición es compleja tanto en ejemplares vivos como en especímenes de museo. Un procedimiento alternativo para disponer de datos morfométricos de las aves puede encontrarse en las bases de datos sistemáticas de fotografías de plumas disponibles en internet. En este trabajo se evalúa si las mediciones de fotografías de plumas presentes en estas bases de datos sirven para obtener estimaciones fiables de la morfometría de las aves.
2. Se recopilaron datos publicados de “*wing area*” para un total de 317 especies de aves, y se pudieron obtener mediciones de plumas en atlas de plumas online de 225 de ellas. A partir de la longitud media de las 5 plumas secundarias distales y la envergadura de las aves, extraída de la bibliografía, se calculó la variable “*lift generation area*” como proxy de la “*wing area*” de cada especie. El ajuste de la variable propuesta a los datos extraídos de la bibliografía se analizó mediante análisis de correlación, explorándose por regresiones lineares las variables explicativas de la falta de ajuste entre especies.
3. La “*lift generation area*” resultó muy informativa de la “*wing area*” para el conjunto de las especies (*R* ^2^>0.98), y las desviaciones observadas entre especies se asociaron fuertemente al tamaño de muestra sobre la que se había calculado originalmente la “*wing area*” (*p* = .001) y en menor medida al tamaño del ave (*p* = .023), pero no al “*aspect ratio*.” Además, se comprobó que el valor medio del desajuste entre “*lift generation area*” y “*wing area*” (13.1%) para el conjunto de las especies es de la misma magnitud que la diferencia que muestran entre fuentes bibliográficas los datos de “*wing area*” de las 64 especies de las que se han publicado dos estimaciones de esta variable (15.3%).
4. Se concluye que el uso de mediciones de plumas procedentes de bases de datos de fotografías disponibles en internet es fiable para estudios de ecología comparada de aves, permitiendo la introducción en ellos de parámetros biomecánicos de muchas más especies que las manejadas hasta la actualidad.

## 1 INTRODUCTION

The comparative study of the morphology and aerodynamics of flying animals has revealed the constraints and physical laws that govern flight, including the existence of general scaling functions between weight, wing loading, and cruising speed across birds (Greenwalt, 1962; Hedenström, 2002; Norberg, 2002; Tennekes, 2009). Wing loading, the ratio of weight to wing area, is central to several aspects of flight such as optimal and minimum flying speed, or maneuverability. For example, the relationship between low wing loading and slow cruising speed in relation to mass is a recurrent finding among animals (from butterflies to vultures and other terrestrial soaring birds), which are strongly limited by the maximum power that they can generate (Tennekes, 2009). In this context, wing area and aspect ratio (the ratio between the square of wingspan and wing area) are two key variables employed in avian comparative morphology, and both rely on the measurement of wing areas (Andrews et al., 2009; Buler et al., 2017; Vágási et al., 2016; Viscor & Fúster, 1987; Warham, 1977). Their systematic analysis has established for instance that the evolutionary pressures to which soaring birds are exposed lead to increases both in wing area and in aspect ratio (larger and narrower wings), after controlling for size and phylogeny (Taylor & Thomas, 2014). It has similarly been shown that for migratory birds, a high wing loading is compensated for by an increased flight speed, although evolutionary constraints compress the range of observed cruising flight speeds among bird species (Alerstam et al., 2007). Such analyses reinforce and refine the historical descriptions of the relationships between bird morphology and flight characteristics, and the classifications of flight styles (Dial, 2003; Pennycuick, 1987; Viscor & Fúster, 1987), supporting them on a quantifiable variable: wing area.

Obtaining wing area data is nonetheless difficult, whether live birds or museum specimens are used. Measuring a live bird may involve extending a wing fully against a surface, so as to draw its outline (Pennycuick, 1989; Warham, 1977) or to take a photograph (Buler et al., 2017), all to be done rapidly without injuring the bird prior to releasing it. Round skins and open‐wing preparations from museums are easier to handle but may have been affected by shrinkage through dehydration (Greenwalt, 1962). The body section between the wings needs to be added to calculate the standard measure of wing area, which adds further imprecision to the measurements since the precise boundary of the proximal end of the wings is difficult to establish (Pennycuick, 1989). In all, the final wing area calculation for any measured individual is inevitably an estimate, irrespective of the method employed. Bearing in mind intraspecific size variation and the small number of measurements available, any wing surface data for a given species are only an approximation to reality. All this notwithstanding, the generalities revealed by comparative studies show the merit of this variable, irrespective of its inherent imprecision.

**FIGURE 1:**
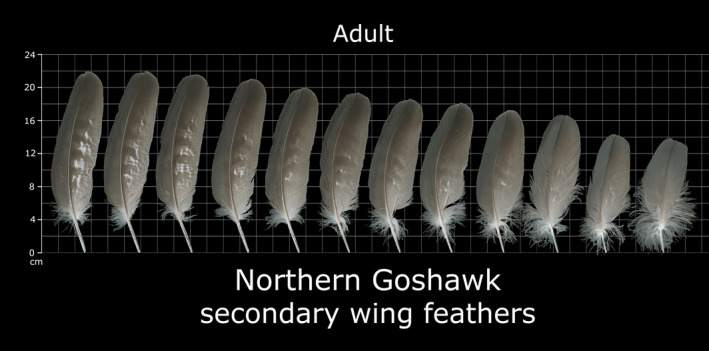
Example of Northern goshawk (*Accipiter* *gentilis*) feather scan with secondaries mounted on a scale grid to allow measurement (USFWS Feather Atlas code: SCAN 61520). The length of first five feathers from the left (F1 to F5) was used to calculate the variable “lift generation area,” a proxy of wing area (see Methods and Figure 2). Image kindly provided by the US Fish and Wildlife Service

Internet images offer an alternative source of avian morphometric data (Featherbase, 2020; USFWS, 2020). These archive high‐definition photographs of feathers alongside a linear scale, allowing precise measurement of each feather (Figure 1). The great merit of databases of this type is their potential scope in terms of the numbers of species and individuals included, given that they benefit from data collection both by scientists and by participants in citizen science, and from carcass material retrieved from various sources (D'Amico et al., 2019; Loss et al., 2015; Wittig et al., 2017). Open‐access databases are rapidly expanding on the Internet and are often of scientific interest given their capacity to gather data from remote locations or resulting from infrequent events, such as encounters with recently dead animals of endangered species (Amano et al., 2016; Périquet et al., 2018; Pocock et al., 2015).

The present study aimed to assess whether web databases of scanned feathers can offer reliable estimates of avian morphometry, with a view to using these in comparative ecology studies. Specifically, we examined whether a combination of bibliographic data on avian wingspans and direct measurements of secondary wing feathers allows an acceptable estimation of wing areas. In such an event, comparative ecology studies will be able to benefit from the mass of data contributed by citizen science, offering reliable morphometric variables for a much greater number of bird species than is currently available in the form of careful measurements of live birds or collection specimens.

## 2 MATERIALS AND METHODS

Wing area data were extracted from the literature, with special emphasis on reviews of multiple bird taxa and, in all cases, on data including the interwing body area, in accordance with Pennycuick (1989). The initial database comprised 317 species in total, with data obtained from Warham (1977), Pennycuick (1987), Brueder and Boldt (2001), Meseguer et al. (2004), Suryan et al. (2008), Agostini et al. (2015), Alerstam et al. (2007), and Vágási et al. (2016). In addition to the wing area data provided, the sample size (*n_i_*) used to calculate this was also noted, with a view to calculating the weighted mean wing area (AvgWA_i_) for those species that featured in more than one source. A sample size of 1 (*n_i_* = 1) was used where the original sample size was not given but this was only included in calculating an average if *n_i_* = 1 was the case in all sources for the species.

**FIGURE 2:**
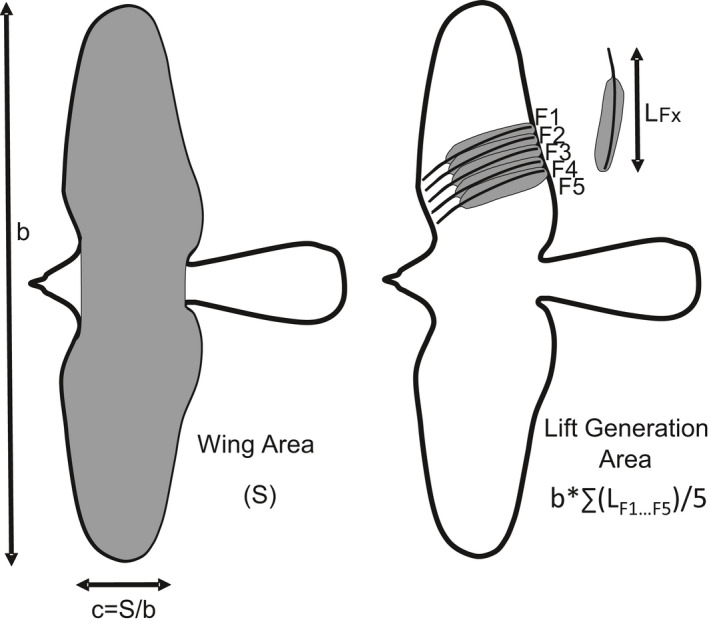
Traditional wing area measurement (left) and that proposed by measuring the lift generation area variable (right), shown on a Northern Goshawk *Accipiter* *gentilis* silhouette. The figure on the right shows the original positions of measured secondary feathers in the wing and a detail of how a collected feather is measured. *S*, wing area (corresponding to shaded area); *b*, wingspan; c, traditional mean chord computation; F1–F5, secondary feathers; *L_Fx_*, length of *x*‐th secondary feather *Fx*

The Featherbase (2020) database has been used as an independent source of avian morphometric data, as well as the United States Fish and Wildlife Service Forensic Laboratory's Feather Atlas (USFWS, 2020) for species not included in the former (Figure 1). The mean length of the 5 distal secondary feathers, as scanned from the database used, was used as a proxy for mean wing chord to estimate the “lift generation area” according to the following equation (Figure 2):

$$\text{Lift}\;\text{generation}\;\text{area}=b*\frac{\sum \left({\text{LF}}_{1}\ldots {\text{LF}}_{5}\right)}{5}$$

where *b* is wingspan, and *LF_1_* to *LF_5_* are the length of secondary feathers 1 to 5. The length of each of the 5 secondaries corresponds in turn to the average of measurements from several individuals. This is included in the Featherbase (2020) database or was calculated from measurements of each individual shown in USFWS (2020). The feather length data were complemented with mean wingspan (*b*) data extracted from general compilations (Hoyo & Sargatal, 1992; SEO, 2020), using the mean of the wingspan range given in the literature.

The lift generation area for each species was then compared with published measurements of wing areas (Figure 2) by Pearson's correlations, both with the original data and with the data log‐transformed, given that wing area comparisons use one or the other of these data. Log‐transformation reduces the weighting of observations of the larger birds, which may be three orders of magnitude greater than those of the smallest (e.g., Great White Pelican *Pelecanus onocrotalus,* 8.5 kg and wing area 0.96 m^2^ vs. Eurasian Wren *Troglodytes* *troglodytes*, 9.6 g and wing area 0.006 m^2^).

The central question underlying this study was answered by determining the proportion of the variance (*R* ^2^) in measured wing area explained by the lift generation area variable obtained from the databases. We also determined whether discrepancies between the values of lift generation area and wing area varied systematically in relation to bird size (mass), wing morphology as measured by aspect ratio (Taylor & Thomas, 2014), or the sample size underlying the wing area estimate. For this purpose, we fitted a linear regression model of lift generation area as a function of the wing area measurement for each species and relativized the absolute values of the residuals as a percentage of the lift generation area predicted for each species. These discrepancy values (arcsin‐transformed) were correlated via Pearson's *R* with variables bird mass, aspect ratio, and sample size; a linear regression model was constructed by means of forward entry with these variables.

At the same time, with a view to establishing the intrinsic variability of traditional wing area measurements, the variation in wing areas between bibliographic sources was analyzed for those species for which two independent data sources were available. This was done by calculating the percentage difference between the published measurements (WAdif) as:

$$\text{WAdif}=100\times \frac{\left|{\text{WA}}_{i1}‐{\text{WA}}_{i2}\right|}{{\text{AvgWA}}_{i}}$$

where WA*_i_*_1_ is the wing area of species i in literature source 1, WA*_i_*_2_ is the wing area of species i in literature source 2, and AvgWA*_i_* is the weighted average of the wing areas calculated here. These data are presented descriptively and analyzed by Pearson's correlations between WAdif and three of these potential explicative variables: total sample size (*n_i_*_1_ + *n_i_*_2_), size of the smallest sample (minimum of *n_i_*_1_ and *n_i_*_2_), and species size (AvgWA*_i_*), given that measuring larger species may involve less error. These correlations were calculated with natural log‐transformed sample sizes and arcsin‐transformed percentages, although the text figures show untransformed data to aid comprehension. Data from sources where the sample size (*n_i_*) was not given were excluded from this analysis.

Statistical analyses used STATISTICA 8.0 (StatSoft, 2007), with a significance level set at *p* < .05.

## 3 RESULTS

**FIGURE 3:**
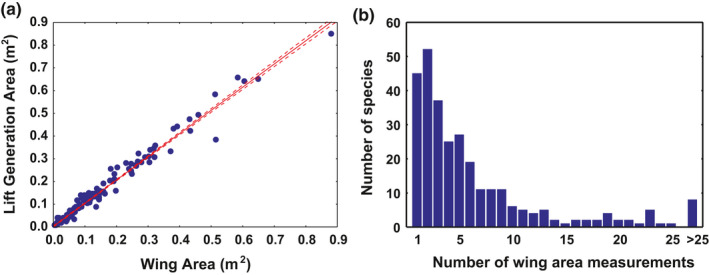
(a) Relationship between mean wing area measured on 225 bird species and the lift generation area variable calculated from digital images of feathers. The linear regression model with its 95% confidence limits is included for information. (b) Sample sizes for the wing area measurements used in this study. The data excluded 26 species found in only one bibliographic source for which sample size *n_i_* was not given

The wing areas compiled here correspond to 317 species. Most are based on measurements of 1–6 individuals (mean ± *SD* 6.52 ± 7.88; median 4), despite being drawn from various sources (Figure 3). Twenty‐six species were excluded from the analysis of wing area intrinsic variability due to the absence of two sources with n_i_. There were images of feather in the web databases for 225 species (71.0%) of the former, enabling their lift generation areas to be calculated and comparisons to be made (Appendix S1). The lift generation area variable proved to be highly informative of wing area for the species set as a whole (Figure 3), whether untransformed (*R* = .991; *p* < .001; *R* ^2^ = .981) or natural log‐transformed (*R* = .991; *p* < .001; *R* ^2^ = .983).

The simple regression model of lift generation area as a function of wing area was highly significant, the absolute value of its residuals being equivalent to 13.1 ± 11.0% of the predicted value. Discrepancies among species were highly variable (range 0.1%–86.7%), and they were negatively correlated with the number of samples used for the wing area estimate (*R *= −.216; *p* = .001; *R* ^2^ = .047) and with bird size (*R *= −.148; *p* = .030; *R* ^2^ = .022), but not with aspect ratio (*R *= −.091; *p* = .183; *R* ^2^ = .008). The stepwise regression model included only the variables sample size (estimate ± *SE* −0.0038 ± 0.0012; *p* = .001) and bird mass (−0.015 ± 0.007; *p* = .023) as explicative of the observed discrepancies.

**FIGURE 4:**
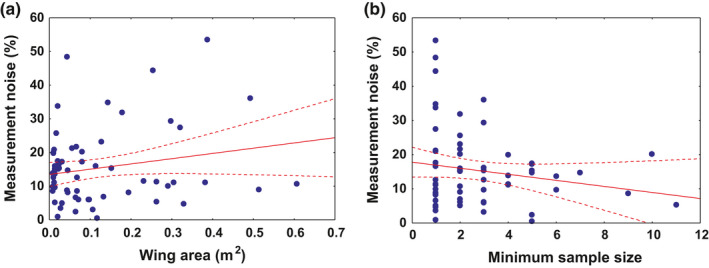
Relationship between variability in wing area measurements in the literature of a particular species (“Measurement noise”), measured as the percentage difference between two independent data sources. (a) Bird size (measured through wing area) and (b) the size of the smallest sample included in the comparison. The linear regression models and their 95% confidence limits are included as a guide in both figures, although neither relationship was significant

The wing area analyses for the 64 species with two independent records in the literature showed that measurements of this variable on live birds or museum specimens have high intrinsic variability (mean ± *SD* WAdif = 15.3 ± 11.1%; range 0.43%–53.4%). The variability observed within published data increases slightly with bird size and is lower for larger samples (Figure 4). Nevertheless, there were no significant correlations with wing area (*R* = .175; *p* = .168; *R* ^2^ = .030), with total size of the original sample (*R *= −0.167; *p* = .188; *R* ^2^ = .028), or with the size of the smallest sample (*R *= −.170; *p* = .179; *R* ^2^ = .029).

## 4 DISCUSSION

First of all, it must be highlighted that the degree of fit of the variable based on digital feather images is more than sufficient for it to be used with equal confidence than traditional wing area measurements for analyses across bird taxa. Its explicative capacity exceeds 98%, far beyond the thresholds that are generally used to consider model variables redundant (Dormann et al., 2013). Furthermore, the ease of acquisition makes data available for a large percentage of species and introduces a very adequate proxy for wing area in studies of comparative avian ecology (Agostini et al., 2015; D'Amico et al., 2019; Santos et al., 2016). Also, the procedure could be used to compute proxy variables for aspect ratio or wing loading that are based on wing area measurements.

The reliability of using lift generation area as a proxy for wing area varies between species, although the differences encountered are largely associated with measurement difficulties rather than with any systematic tendencies in the morphology of particular bird groups. The mean discrepancy between both variables is 13%, very similar to that found between independent measurements of wing areas of live birds or museum specimens (15%; see below). It is smaller in cases where multiple wing area measurements are available and also for the larger birds considered. A review of species for which larger discrepancies between the two variables were found revealed some exceptional cases, such as the Hawfinch *Coccothraustes coccothraustes* with its very characteristic blunt secondary feathers and which generates the largest positive discrepancy in our database (estimated lift generation area vs. expected +87%). There are also species in which the transition between the wing and the body is very gradual as a result of having very long proximal secondaries, which may result in difficulties in deciding the exact position of the wingbase, leading to high variation in estimating the interwing area below the body (Pennycuick, 1989). A relatively large negative discrepancy has been found among some of these species (e.g., −56% in Long‐tailed Duck *Clangula hyemalis* and −40% in Common Sandpiper *Actitis hypoleucos*), although a large positive discrepancy has been found in others that also have similar proximal secondaries. (e.g., +43% in Goosander/Common Merganser *Mergus* *merganser*). In fact, a complementary analysis carried out at the Order level showed that the explanatory power of lift generation area for wing area was high in all cases (*R* ^2^ in the range .938–.999).

From all this, it emerges that the length of the distal secondaries is a good proxy for mean wing chord across species, although it may be less precise in certain cases. The decision to use the average of five secondaries was taken at the beginning of the study after a first review of scans from several species, and the analysis of collected data (not presented in Section 3) shows that: (a) secondary feather length decreases from s1 to s5 in most species, but there are several counterexamples (e.g., *Apus* spp., *Ardea* *alba*), and (b) average discrepancies across species of individual secondary feather lengths with respect to the s1–s5 average reach a minimum for s3 (s1, +3.1%; s2, +2.1%; s3, +0.34%; s4, −1.6%; and s5, −3.9%). However, the difference in length among s1–s5 secondaries for some species may reach up to 20%–30%, as it happens in some Galliformes with a small s1 feather (e.g., *Phasianus colchicus,* 30.3%; and *Perdix perdix,* 27.8%). Since this study covers a limited number of species, it seems advisable to use the average of s1–s5 until we have a deeper knowledge of patterns of variability in secondaries among bird species. The use of s3 would be a conservative option in cases where it is not possible to measure several feathers, though the observed differences with the average were in the range between −3.3% and +5.8%.

The methodology explored here has the principal advantages that it enables access to data from a great number of species and individuals and that it is simple to measure. At the time when the present data were extracted, the two databases used contained respectively feather images of 1513 species (Featherbase, 2020) and 422 species (USFS, 2020), with approximately 19% of species in common. Together, these numbers are greater even than those used in wing area compilations (e.g., *n* = 450 species in Taylor & Thomas, 2014, and *n* = 150 in Vágási et al., 2016), though they are still a small fraction of all bird species (Hoyo & Sargatal, 1992; Pigot et al., 2020). This makes them more useful for comparative studies and allows very broad application. Hence, for present purposes, the databases included 71% of those species for which wing area data are available, and a study on bird mortality along highspeed train lines (Malo et al., 2016) was able to find data for 86% of the relevant species even though published wing area data only included 34% of them (J. E. Malo et al. unpublished). The breadth of the existing photograph databases is enhanced by their great capacity to expand their coverage of species and individuals, thanks to the contributions of citizen science (Pocock et al., 2018) and to the possibility of taking advantage of dead birds found at buildings, roads, windfarms, and other human infrastructures (Santos et al., 2016; Wittig et al., 2017). Moreover, citizen science is known to provide data overrepresenting large and unfrequent species (Périquet et al., 2018), an interesting point since lift generation area is more accurate for larger species and wing area measurements are less reliable for those with few measurements or large body size (see below). In the medium term, the number of species and individuals included should be sufficiently large for the data also to be used to facilitate intraspecific studies (García Antón et al., 2018; Swaddle & Lockwood, 2003). Similarly, feather photographs may also enable estimation of other indices of wing structure based on the measurement of primaries and secondaries (e.g., hand wing index and Kipp's index) that are often used in avian comparative ecology studies (Claramunt et al., 2012; Lockwood et al., 1998; Phillips et al., 2018; Pigot et al., 2020).

Once feather images have been taken in a standardized way, feather measurement is straightforward and potentially applicable to new images or to material from less specialized repositories (e.g., Slater Museum, 2020). Photogrammetry of digital images is used routinely in diverse areas of zoology and ecology (Breuer et al., 2007; Mendes et al., 2016; Munge & Athreya, 2020; Rycroft et al., 2013), its simplicity and precision having been demonstrated repeatedly (Ireland et al., 2006; Muñoz‐Muñoz & Perpiñán, 2010; Ortega‐Ortiz et al., 2018). Hence, bearing in mind the anticipated digitalization of museum collections (Medina et al., 2020) and the expected addition of new material to the databases, we may be confident that images from which to obtain precise data on feather dimensions and variability will be available in the medium term for a large proportion of avian species. In the meanwhile, the procedure presented here can be also applied with small errors to feathers directly measured from open‐wing preparations and bird round skins preserved in collections.

In relation to the precision of data, it must be stressed that the intrinsic variability of wing area measurements is considerable, as shown here by the comparisons of data for species for which two independent data sources were available. The measurements repeatedly show variation between independent data sources of a particular species of 15%, often over 25%. This variability may be due in part to intraspecific variation, such as often occurs between sexes or within species that have an extensive geographical range that includes both sedentary and migratory populations, or inhabit diverse environments (Andrews et al., 2009; Baldwin et al., 2010; García Antón et al., 2018). Such intrinsic variability also affects the feather measurements considered here, so the representativity of feather measurement data will increase with sample size.

Nevertheless, a large part of the variability in wing area measurements is undoubtedly due to the difficulty of measuring whole specimens, either from collections or as live individuals (Buler et al., 2017; Warham, 1977). In any event, the repeatability of measurements is rather loosely linked with bird size and sample size. It is notable that a variability of 15% remains even among measurements of 4–7 individuals; more than 10 individuals are probably needed to produce relatively stable estimates. Bearing in mind the sample sizes used here, the imprecision of most bibliographic data probably exceeds 15%, no doubt reflecting the mix of different ages, sexes, or origins. Furthermore, and contrary to expectations, the larger species show the greater variation in measurements between sources, possibly because they are also based on smaller sample sizes, many are based on museum specimens, and they are particularly difficult to measure precisely in the field (Warham, 1977). Thus, the direct measurement of secondary feathers from collections and the calculation of the wing loading area can also be a good alternative here.

To conclude, the proposed method for estimating a proxy of wing area of birds by means of feather measurements, based on digital images taken from databases available on the Internet, proves to be reliable. It will facilitate the incorporation of trustworthy biomechanical parameters for many species into studies of avian comparative morphology.

## CONFLICT OF INTEREST

The authors declare the absence of conflicts of interest.

## AUTHOR CONTRIBUTIONS

**Juan E. Malo:** Conceptualization (lead); Data curation (equal); Formal analysis (equal); Methodology (equal); Writing‐original draft (equal); Writing‐review & editing (equal). **Cristina Mata:** Data curation (equal); Formal analysis (equal); Methodology (equal); Writing‐original draft (equal); Writing‐review & editing (equal).

## Supporting information

Appendix S1

## Data Availability

The dataset is available at the Open Free Digital Repository e‐cienciaDatos with doi accession number: https://doi.org/10.21950/ZYAOU6.
