# Supplementary material for: Web databases of feather photographs are useful tools for avian morphometry studies
Source: Ecol Evol. 2021 Jun 2;11(12):7677–84. doi: 10.1002/ece3.7600 (PMC8216968; doi:10.1002/ece3.7600)
Supplement: Supplementary file 1 — Appendix S1 [file ECE3-11-7677-s001.docx]

Appendix 1. Main biometric data of birds used in the paper. Bird mass, wing area and sample size come from the original bibliographic sources for wing areas. Wing span was obtained from general compilations and lift generation area calculated as explained in the text. N/A, data not available in the original source.

| **Species** | **Mass (kg)** | **Wing span (m)** | **Wing area (m^2^)** | **Sample size** | **Lift generation area (m^2^)** |
| --- | --- | --- | --- | --- | --- |
| *Accipiter gentilis* | 0.935 | 1.055 | 0.1928 | 8 | 0.1984 |
| *Accipiter nisus* | 0.257 | 0.685 | 0.0798 | 53 | 0.0844 |
| *Acrocephalus arundinaceus* | 0.030 | 0.265 | 0.0171 | 5 | 0.0160 |
| *Acrocephalus palustris* | 0.012 | 0.195 | 0.0081 | 5 | 0.0086 |
| *Acrocephalus schoenobaenus* | 0.011 | 0.190 | 0.0083 | 5 | 0.0080 |
| *Acrocephalus scirpaceus* | 0.012 | 0.190 | 0.0083 | 4 | 0.0083 |
| *Actitis hypoleucos* | 0.058 | 0.335 | 0.0244 | N/A | 0.0164 |
| *Aegithalos caudatus* | 0.009 | 0.175 | 0.0073 | 8 | 0.0079 |
| *Alauda arvensis* | 0.039 | 0.330 | 0.0213 | 6 | 0.0219 |
| *Alca torda* | 0.620 | 0.655 | 0.0462 | 3 | 0.0448 |
| *Alcedo atthis* | 0.036 | 0.250 | 0.0128 | 7 | 0.0127 |
| *Alectoris chukar* | 0.560 | 0.495 | 0.0582 | N/A | 0.0526 |
| *Anas acuta* | 1.024 | 0.830 | 0.0879 | 2 | 0.0865 |
| *Anas crecca* | 0.327 | 0.560 | 0.0462 | 10 | 0.0442 |
| *Anas penelope* | 0.783 | 0.780 | 0.0814 | 11 | 0.0796 |
| *Anas platyrhynchos* | 1.112 | 0.880 | 0.1066 | 27 | 0.1082 |
| *Anas querquedula* | 0.353 | 0.615 | 0.0566 | 2 | 0.0469 |
| *Anser albifrons* | 2.582 | 1.450 | 0.1835 | 1 | 0.2527 |
| *Anser anser* | 3.326 | 1.635 | 0.3079 | 6 | 0.3393 |
| *Anser fabalis* | 3.035 | 1.585 | 0.2675 | 1 | 0.2869 |
| *Anthus pratensis* | 0.016 | 0.240 | 0.0123 | 4 | 0.0129 |
| *Anthus spinoletta* | 0.024 | 0.270 | 0.0164 | 2 | 0.0167 |
| *Anthus trivialis* | 0.023 | 0.260 | 0.0139 | 5 | 0.0149 |
| *Apus apus* | 0.040 | 0.450 | 0.0168 | 13 | 0.0193 |
| *Apus melba* | 0.087 | 0.570 | 0.0312 | 2 | 0.0287 |
| *Aquila chrysaetos* | 4.166 | 2.050 | 0.6070 | 13 | 0.6406 |
| *Aquila pomarina* | 1.496 | 1.500 | 0.5152 | 2 | 0.3831 |
| *Ardea alba* | 0.874 | 1.575 | 0.4357 | 1 | 0.4219 |
| *Ardea cinerea* | 1.441 | 1.650 | 0.3822 | 4 | 0.4302 |
| *Arenaria interpres* | 0.111 | 0.460 | 0.0252 | 11 | 0.0280 |
| *Asio flammeus* | 0.325 | 1.000 | 0.1638 | 3 | 0.1460 |
| *Asio otus* | 0.299 | 0.920 | 0.1372 | 16 | 0.1329 |
| *Athene noctua* | 0.164 | 0.525 | 0.0611 | 6 | 0.0543 |
| *Aythya ferina* | 0.823 | 0.710 | 0.0649 | 5 | 0.0655 |
| *Aythya fuligula* | 0.698 | 0.685 | 0.0563 | 4 | 0.0623 |
| *Aythya marila* | 0.931 | 0.755 | 0.0621 | 1 | 0.0690 |
| *Bombycilla garrulus* | 0.054 | 0.340 | 0.0201 | 2 | 0.0215 |
| *Botaurus stellaris* | 1.229 | 1.300 | 0.2628 | 3 | 0.2696 |
| *Branta canadensis* | 3.628 | 1.500 | 0.3717 | 4 | 0.3318 |
| *Branta bernicla* | 1.306 | 1.110 | 0.1129 | 3 | 0.1473 |
| *Bubo bubo* | 2.686 | 1.640 | 0.3931 | 2 | 0.4395 |
| *Bucephala clanga* | 0.901 | 0.695 | 0.0598 | 2 | 0.0646 |
| *Buteo buteo* | 0.831 | 1.210 | 0.2628 | 25 | 0.2671 |
| *Buteo lagopus* | 0.950 | 1.350 | 0.3214 | 8 | 0.3054 |
| *Callidris alpina* | 0.054 | 0.405 | 0.0156 | 23 | 0.0194 |
| *Callidris canutus* | 0.128 | 0.500 | 0.0286 | 19 | 0.0318 |
| *Calonectris diomedea* | 0.946 | 1.225 | 0.1114 | 3 | 0.1337 |
| *Caprimulgus europaeus* | 0.067 | 0.260 | 0.0405 | 1 | 0.0217 |
| *Carduelis carduelis* | 0.016 | 0.233 | 0.0099 | 9 | 0.0111 |
| *Carduelis chloris* | 0.028 | 0.260 | 0.0123 | 6 | 0.0138 |
| *Carduelis flammea* | 0.011 | 0.225 | 0.0083 | N/A | 0.0099 |
| *Carduelis spinus* | 0.014 | 0.215 | 0.0080 | 19 | 0.0090 |
| *Carpodacus erythrinus* | 0.024 | 0.250 | 0.0127 | 4 | 0.0128 |
| *Cecropis daurica* | 0.022 | 0.330 | 0.0172 | 6 | 0.0169 |
| *Certhia familiaris* | 0.009 | 0.193 | 0.0089 | 2 | 0.0092 |
| *Charadrius hiaticula* | 0.064 | 0.380 | 0.0179 | 9 | 0.0180 |
| *Chroicocephalus ridibundus* | 0.284 | 1.050 | 0.0980 | 30 | 0.1172 |
| *Ciconia ciconia* | 3.440 | 1.990 | 0.5142 | 5 | 0.5820 |
| *Cinclus cinclus* | 0.062 | 0.275 | 0.0143 | 3 | 0.0167 |
| *Circaetus gallicus* | 1.700 | 1.675 | 0.4320 | 2 | 0.4742 |
| *Circus aeruginosus* | 0.613 | 1.275 | 0.2040 | 11 | 0.2594 |
| *Circus cyaneus* | 0.417 | 1.075 | 0.1797 | 5 | 0.2030 |
| *Clangula hyemalis* | 0.874 | 0.310 | 0.0669 | 2 | 0.0312 |
| *Coccothraustes coccothraustes* | 0.057 | 0.660 | 0.0189 | 5 | 0.0402 |
| *Coloeus monedula* | 0.246 | 0.685 | 0.0711 | 8 | 0.0873 |
| *Columba livia* | 0.355 | 0.665 | 0.0701 | 4 | 0.0730 |
| *Columba oenas* | 0.295 | 0.660 | 0.0532 | 1 | 0.0697 |
| *Columba palumbus* | 0.450 | 0.775 | 0.0842 | 3 | 0.1010 |
| *Coracias garrulus* | 0.146 | 0.695 | 0.0661 | 4 | 0.0756 |
| *Corvus corax* | 1.100 | 1.275 | 0.2472 | 1 | 0.2783 |
| *Corvus cornix* | 0.570 | 0.920 | 0.1479 | 2 | 0.1564 |
| *Corvus corone* | 0.525 | 0.920 | 0.1378 | 5 | 0.1690 |
| *Corvus frugilegus* | 0.471 | 0.875 | 0.1334 | 4 | 0.1441 |
| *Crex crex* | 0.156 | 0.495 | 0.0407 | 5 | 0.0392 |
| *Cuculus canorus* | 0.112 | 0.570 | 0.0612 | 5 | 0.0523 |
| *Cyanistes caeruleus* | 0.011 | 0.188 | 0.0095 | 6 | 0.0088 |
| *Cygnus columbianus* | 6.637 | 1.950 | 0.4608 | 11 | 0.4910 |
| *Cygnus olor* | 10.597 | 2.230 | 0.6504 | 9 | 0.6488 |
| *Delichon urbicum* | 0.015 | 0.280 | 0.0111 | 6 | 0.0126 |
| *Dendrocopos major* | 0.077 | 0.410 | 0.0388 | 5 | 0.0358 |
| *Diomedea immutabilis* | 2.931 | 1.990 | 0.3051 | 24 | 0.2840 |
| *Dryocopus martius* | 0.321 | 0.660 | 0.1088 | 2 | 0.1033 |
| *Emberiza calandra* | 0.049 | 0.280 | 0.0178 | 2 | 0.0182 |
| *Emberiza cia* | 0.023 | 0.243 | 0.0151 | 3 | 0.0152 |
| *Emberiza citrinella* | 0.030 | 0.263 | 0.0176 | 2 | 0.0158 |
| *Emberiza schoeniclus* | 0.018 | 0.245 | 0.0135 | 4 | 0.0137 |
| *Erithacus rubecula* | 0.018 | 0.210 | 0.0110 | 6 | 0.0110 |
| *Falco biarmicus* | 0.700 | 1.000 | 0.1418 | N/A | 0.1440 |
| *Falco cherrug* | 0.966 | 1.150 | 0.1606 | 1 | 0.1898 |
| *Falco naumanni* | 0.141 | 0.690 | 0.0611 | 2 | 0.0658 |
| *Falco peregrinus* | 0.743 | 1.010 | 0.1285 | 21 | 0.1335 |
| *Falco subbuteo* | 0.224 | 0.785 | 0.0679 | 7 | 0.0750 |
| *Falco tinnunculus* | 0.194 | 0.730 | 0.0803 | 16 | 0.0808 |
| *Falco vespertinus* | 0.159 | 0.705 | 0.0684 | 4 | 0.0662 |
| *Ficedula albicollis* | 0.013 | 0.235 | 0.0111 | 3 | 0.0121 |
| *Ficedula hypoleuca* | 0.014 | 0.228 | 0.0104 | 6 | 0.0113 |
| *Fratercula arctica* | 0.398 | 0.550 | 0.0369 | 10 | 0.0353 |
| *Fringilla coelebs* | 0.023 | 0.265 | 0.0137 | 23 | 0.0153 |
| *Fringilla montifringilla* | 0.024 | 0.250 | 0.0133 | 20 | 0.0138 |
| *Fulica atra* | 0.836 | 0.750 | 0.0903 | 2 | 0.0867 |
| *Fulmarus glacialis* | 0.789 | 1.070 | 0.1148 | 6 | 0.1077 |
| *Galerida cristata* | 0.043 | 0.335 | 0.0240 | 1 | 0.0235 |
| *Gallinago gallinago* | 0.119 | 0.455 | 0.0445 | 7 | 0.0290 |
| *Gallinula chloropus* | 0.305 | 0.530 | 0.0514 | 2 | 0.0527 |
| *Garrulus glandarius* | 0.165 | 0.560 | 0.0654 | 14 | 0.0725 |
| *Gavia arctica* | 2.811 | 1.200 | 0.1447 | 2 | 0.1410 |
| *Gavia stellata* | 1.505 | 1.110 | 0.0890 | 1 | 0.1383 |
| *Grus grus* | 5.614 | 2.010 | 0.5855 | 2 | 0.6565 |
| *Haematopus ostralegus* | 0.523 | 0.775 | 0.0810 | 4 | 0.0815 |
| *Haliaeetus albicilla* | 4.967 | 2.200 | 0.8824 | 4 | 0.8481 |
| *Hippolais icterina* | 0.013 | 0.220 | 0.0110 | 5 | 0.0109 |
| *Hippolais polyglotta* | 0.011 | 0.220 | 0.0061 | N/A | 0.0102 |
| *Hirundo rupestris* | 0.019 | 0.330 | 0.0116 | 2 | 0.0162 |
| *Hirundo rustica* | 0.017 | 0.330 | 0.0147 | 13 | 0.0163 |
| *Ixobrychus minutus* | 0.118 | 0.550 | 0.0440 | 5 | 0.0476 |
| *Jynx torquilla* | 0.035 | 0.260 | 0.0156 | 5 | 0.0151 |
| *Lanius collurio* | 0.028 | 0.255 | 0.0149 | 5 | 0.0146 |
| *Lanius minor* | 0.047 | 0.330 | 0.0220 | 3 | 0.0225 |
| *Larus argentatus* | 1.041 | 1.400 | 0.1955 | 12 | 0.2311 |
| *Larus cachinnans* | 1.150 | 1.410 | 0.2496 | N/A | 0.2322 |
| *Larus canus* | 0.411 | 1.150 | 0.1246 | 3 | 0.1458 |
| *Larus fuscus* | 0.719 | 1.340 | 0.1934 | 14 | 0.2091 |
| *Larus marinus* | 1.615 | 1.550 | 0.2907 | 11 | 0.3071 |
| *Larus michahellis* | 1.154 | 1.440 | 0.2474 | 1 | 0.2444 |
| *Limosa lapponica* | 0.318 | 0.750 | 0.0520 | 1 | 0.0593 |
| *Linaria cannabina* | 0.017 | 0.230 | 0.0110 | 6 | 0.0106 |
| *Locustella luscinioides* | 0.014 | 0.195 | 0.0081 | 7 | 0.0081 |
| *Loxia curvirostra* | 0.041 | 0.285 | 0.0133 | 1 | 0.0152 |
| *Lullula arborea* | 0.027 | 0.285 | 0.0164 | 1 | 0.0178 |
| *Luscinia luscinia* | 0.024 | 0.180 | 0.0135 | 3 | 0.0104 |
| *Luscinia megarhynchos* | 0.018 | 0.245 | 0.0136 | 7 | 0.0131 |
| *Melanitta fusca* | 1.743 | 0.880 | 0.1010 | 1 | 0.1062 |
| *Melanitta nigra* | 0.990 | 0.770 | 0.0679 | 1 | 0.0814 |
| *Mergus merganser* | 1.489 | 0.895 | 0.0767 | 2 | 0.1155 |
| *Mergus serrator* | 1.004 | 0.745 | 0.0678 | 2 | 0.0777 |
| *Merops apiaster* | 0.057 | 0.465 | 0.0278 | 2 | 0.0330 |
| *Milvus migrans* | 0.845 | 1.375 | 0.2803 | 2 | 0.2836 |
| *Milvus milvus* | 1.075 | 1.620 | 0.3248 | 3 | 0.3583 |
| *Monticola saxatilis* | 0.050 | 0.350 | 0.0204 | N/A | 0.0238 |
| *Morus bassanus* | 3.010 | 1.725 | 0.2620 | 1 | 0.2712 |
| *Motacilla alba* | 0.021 | 0.280 | 0.0124 | 12 | 0.0152 |
| *Motacilla cinérea* | 0.018 | 0.260 | 0.0114 | 8 | 0.0127 |
| *Motacilla flava* | 0.018 | 0.250 | 0.0115 | 2 | 0.0126 |
| *Muscicapa striata* | 0.016 | 0.243 | 0.0136 | 7 | 0.0129 |
| *Nucifraga caryocatactes* | 0.173 | 0.550 | 0.0584 | 2 | 0.0701 |
| *Numenius arquata* | 0.794 | 0.975 | 0.1182 | 2 | 0.1186 |
| *Numenius phaeopus* | 0.383 | 0.825 | 0.1365 | 1 | 0.0871 |
| *Nycticorax nycticorax* | 0.763 | 0.950 | 0.1589 | 2 | 0.1543 |
| *Oceanodroma leucorhoa* | 0.039 | 0.465 | 0.0237 | 32 | 0.0291 |
| *Oenanthe oenanthe* | 0.024 | 0.290 | 0.0145 | 8 | 0.0178 |
| *Oriolus oriolus* | 0.079 | 0.450 | 0.0272 | 1 | 0.0367 |
| *Otus scops* | 0.092 | 0.505 | 0.0491 | 3 | 0.0466 |
| *Pandion haliaetus* | 1.578 | 1.605 | 0.3201 | 22 | 0.3456 |
| *Panurus biarmicus* | 0.014 | 0.170 | 0.0076 | 7 | 0.0071 |
| *Parus ater* | 0.009 | 0.190 | 0.0080 | 7 | 0.0084 |
| *Parus major* | 0.019 | 0.240 | 0.0112 | 19 | 0.0134 |
| *Passer domesticus* | 0.028 | 0.233 | 0.0124 | 2 | 0.0121 |
| *Passer hispaniolensis* | 0.024 | 0.245 | 0.0124 | 5 | 0.0126 |
| *Passer montanus* | 0.021 | 0.210 | 0.0095 | 4 | 0.0100 |
| *Perdix perdix* | 0.406 | 0.465 | 0.0514 | 3 | 0.0427 |
| *Pernis apivorus* | 0.778 | 1.240 | 0.2471 | 10 | 0.2418 |
| *Phalacrocorax carbo* | 2.168 | 1.450 | 0.2321 | 13 | 0.2785 |
| *Phalaropus fulicarius* | 0.054 | 0.385 | 0.0196 | 6 | 0.0207 |
| *Phalaropus lobatus* | 0.033 | 0.320 | 0.0146 | 4 | 0.0156 |
| *Phasianus colchicus* | 1.135 | 0.800 | 0.1447 | 5 | 0.1200 |
| *Philomachus pugnax* | 0.114 | 0.540 | 0.0388 | 9 | 0.0365 |
| *Phoenicopterus ruber* | 3.053 | 1.550 | 0.2715 | 1 | 0.3230 |
| *Phoenicurus ochruros* | 0.017 | 0.245 | 0.0138 | 6 | 0.0144 |
| *Phoenicurus phoenicurus* | 0.015 | 0.230 | 0.0111 | 2 | 0.0122 |
| *Phylloscopus collybita* | 0.008 | 0.180 | 0.0075 | 5 | 0.0077 |
| *Phylloscopus sibilatrix* | 0.009 | 0.215 | 0.0097 | 5 | 0.0104 |
| *Phylloscopus trochilus* | 0.009 | 0.190 | 0.0086 | 3 | 0.0083 |
| *Pica pica* | 0.206 | 0.560 | 0.0681 | 5 | 0.0763 |
| *Picus canus* | 0.137 | 0.390 | 0.0416 | 2 | 0.0394 |
| *Picus viridis* | 0.176 | 0.410 | 0.0537 | 3 | 0.0467 |
| *Platalea leucorodia* | 1.857 | 1.275 | 0.2415 | 2 | 0.2542 |
| *Plegadis falcinellus* | 0.566 | 0.875 | 0.0986 | 1 | 0.1386 |
| *Pluvialis squatarola* | 0.219 | 0.565 | 0.0420 | 6 | 0.0389 |
| *Podiceps cristatus* | 0.674 | 0.875 | 0.0683 | 1 | 0.0835 |
| *Poecile palustris* | 0.011 | 0.188 | 0.0091 | 8 | 0.0090 |
| *Porzana parva* | 0.050 | 0.365 | 0.0136 | 1 | 0.0194 |
| *Porzana porzana* | 0.083 | 0.395 | 0.0250 | 3 | 0.0271 |
| *Prunella modularis* | 0.020 | 0.200 | 0.0093 | 18 | 0.0101 |
| *Ptynoprogne rupestris* | 0.024 | 0.330 | 0.0113 | N/A | 0.0159 |
| *Pyrrhula pyrrhula* | 0.022 | 0.255 | 0.0141 | 8 | 0.0153 |
| *Rallus aquaticus* | 0.112 | 0.415 | 0.0335 | 3 | 0.0312 |
| *Regulus ignicapilla* | 0.006 | 0.145 | 0.0049 | 2 | 0.0054 |
| *Regulus regulus* | 0.006 | 0.145 | 0.0052 | 2 | 0.0053 |
| *Remiz pendulinus* | 0.009 | 0.165 | 0.0069 | 1 | 0.0069 |
| *Riparia riparia* | 0.014 | 0.280 | 0.0108 | 9 | 0.0125 |
| *Rissa tridactyla* | 0.398 | 1.065 | 0.0972 | 9 | 0.1178 |
| *Saxicola rubetra* | 0.017 | 0.225 | 0.0115 | 9 | 0.0111 |
| *Saxicola rubicola* | 0.015 | 0.195 | 0.0090 | 4 | 0.0093 |
| *Scolopax rusticola* | 0.310 | 0.600 | 0.0681 | 1 | 0.0643 |
| *Serinus serinus* | 0.011 | 0.215 | 0.0090 | 2 | 0.0099 |
| *Sitta europaea* | 0.023 | 0.245 | 0.0148 | 7 | 0.0145 |
| *Somateria mollissima* | 2.015 | 1.000 | 0.1310 | 2 | 0.1461 |
| *Somateria spectabilis* | 1.591 | 0.940 | 0.1080 | 1 | 0.1179 |
| *Stercorarius longicaudus* | 0.297 | 1.100 | 0.0891 | 4 | 0.1257 |
| *Stercorarius parasiticus* | 0.414 | 1.175 | 0.1174 | 21 | 0.1354 |
| *Sterna hirundo* | 0.120 | 0.775 | 0.0587 | 3 | 0.0596 |
| *Sterna paradisaea* | 0.110 | 0.805 | 0.0571 | 1 | 0.0586 |
| *Streptopelia decaocto* | 0.149 | 0.510 | 0.0473 | 4 | 0.0526 |
| *Streptotelia senegalensis* | 0.088 | 0.425 | 0.0304 | N/A | 0.0357 |
| *Strix aluco* | 0.475 | 0.885 | 0.1980 | 3 | 0.1573 |
| *Strix uralensis* | 0.785 | 1.290 | 0.3042 | 3 | 0.3092 |
| *Sturnus vulgaris* | 0.085 | 0.395 | 0.0251 | 17 | 0.0285 |
| *Sylvia atricapilla* | 0.017 | 0.215 | 0.0111 | 5 | 0.0105 |
| *Sylvia borin* | 0.018 | 0.225 | 0.0109 | 3 | 0.0109 |
| *Sylvia communis* | 0.015 | 0.205 | 0.0108 | 3 | 0.0100 |
| *Sylvia curruca* | 0.011 | 0.185 | 0.0090 | 5 | 0.0085 |
| *Sylvia nisoria* | 0.023 | 0.250 | 0.0137 | 2 | 0.0145 |
| *Tetrastes bonasia* | 0.429 | 0.510 | 0.0583 | 1 | 0.0558 |
| *Tringa glaréola* | 0.067 | 0.370 | 0.0203 | 5 | 0.0204 |
| *Tringa nebularia* | 0.181 | 0.690 | 0.0431 | 3 | 0.0531 |
| *Troglodytes troglodytes* | 0.010 | 0.150 | 0.0056 | 9 | 0.0058 |
| *Turdus iliacus* | 0.061 | 0.338 | 0.0223 | 35 | 0.0236 |
| *Turdus merula* | 0.113 | 0.365 | 0.0304 | 6 | 0.0320 |
| *Turdus philomelos* | 0.068 | 0.345 | 0.0221 | 23 | 0.0252 |
| *Turdus viscivorus* | 0.114 | 0.445 | 0.0333 | 2 | 0.0397 |
| *Turdus pilaris* | 0.106 | 0.405 | 0.0322 | 12 | 0.0344 |
| *Tyto alba* | 0.350 | 0.875 | 0.1452 | 3 | 0.1294 |
| *Upupa epops* | 0.061 | 0.460 | 0.0431 | 3 | 0.0458 |
| *Uria aalgae* | 0.950 | 0.685 | 0.0544 | 3 | 0.0438 |
| *Vanellus vanellus* | 0.219 | 0.695 | 0.0744 | 2 | 0.0822 |
